# Serum butyraldehyde levels are associated with abdominal aortic calcification: a population-based study

**DOI:** 10.1186/s40001-025-03827-2

**Published:** 2026-01-06

**Authors:** Na Liu, Yun Li, Xiaoshuang Xu, Yi Wang, Ying Zhang, Wenjuan Li, Maiye Zhang, Jie Zhou

**Affiliations:** 1 Department of Endocrinology, Xijing Hospital, The Fourth Military Medical University, Xi’an, 710032 Shaanxi China; 2 Department of Gastroenterology Department, The Fourth Hospital of Changsha, Changsha, 410017 Hunan China

**Keywords:** Abdominal aortic calcification, Aldehyde, Butyraldehyde, NHANES

## Abstract

**Background:**

Abdominal aortic calcification (AAC) is a strong predictor of cardiovascular morbidity but lacks effective interventions. Aldehydes, a diverse class of environmental and endogenous compounds, have been implicated in various chronic diseases, yet their individual and combined effects on vascular calcification remain unclear.

**Methods:**

This study investigated associations between six serum aldehydes (benzaldehyde, butyraldehyde, heptanaldehyde, hexanaldehyde, isopentanaldehyde, propanaldehyde) and AAC in a nationally representative sample of US adults from the 2013–2014 NHANES. AAC was measured via lateral lumbar spine DXA, and serum aldehydes were quantified using gas chromatography-mass spectrometry. Analyses using weighted logistic regression and quantile g-computation (qgcomp) models, adjusted for demographic and metabolic confounders, revealed the associations between aldehydes and AAC.

**Results:**

This study included 1208 NHANES participants. Each log₂-unit increase in butyraldehyde concentration was associated with a 36.7% lower AAC risk (adjusted OR = 0.633, 95% CI 0.436–0.930), and individuals in the highest tertile of exposure had a 46% reduced risk compared to those in the lowest tertile. After FDR correction for multiple comparisons, butyraldehyde exhibited the strongest association with AAC among the aldehydes examined (FDR-adjusted *p* = 0.096). None of the other aldehydes showed significant associations with AAC in fully adjusted models. In the mixture analysis, butyraldehyde also demonstrated the strongest inverse association with AAC (β = −0.442). The inverse association between butyraldehyde and AAC was more pronounced in individuals with klotho deficiency.

**Conclusion:**

These findings indicate that butyraldehyde is inversely associated with AAC, not only highlighting its potential role in vascular calcification but also suggesting the necessity of aldehyde-specific assessments in cardiovascular risk assessment. Besides, butyraldehyde may serve as a promising candidate for therapeutic development in high-risk populations.

## Introduction

Arterial calcification is a systemic pathological process characterized by the deposition of calcium within the arterial walls, leading to increased arterial stiffness and elevated pulse pressure, which in turn impairs microvascular function. Among the various forms of vascular calcification, abdominal aortic calcification (AAC) is the most prevalent subtype. It is closely associated with atherosclerosis and serves as a strong predictor of adverse cardiovascular outcomes [1]. Non-invasive imaging techniques, such as lateral lumbar spine radiography and dual-energy X-ray absorptiometry, provide convenient and efficient tools for assessing AAC [2, 3]. However, there are currently no effective interventions to slow or reverse the progression of AAC. Thus, identifying risk factors or protective factors influencing AAC development is of critical importance for the prevention and management of cardiovascular disease.

Aldehydes, a class of organic compounds containing the –HC = O functional group, are ubiquitously present in the environment and are also generated endogenously within the human body [4]. Accumulating evidence indicates that aldehydes play significant roles in the pathogenesis of various chronic diseases, including atherosclerosis, neurodegenerative disorders [5, 6], and diabetes [7, 8]. Endogenous aldehydes are mainly produced through lipid peroxidation, oxidative stress, and metabolic pathways [9]. For example, 4-hydroxynonenal (4-HNE) and malondialdehyde, both byproducts of lipid peroxidation, are known to induce endothelial dysfunction by modifying low-density lipoprotein and promoting foam cell formation [10].

Interestingly, not all aldehydes are harmful. Certain naturally occurring aldehydes have demonstrated protective cardiovascular properties. Protocatechuic aldehyde, derived from plant, can enhance nitric oxide production and inhibit atherosclerosis [11]. Similarly, cinnamaldehyde, which is a major bioactive component found in cinnamon, exerts antioxidative and anti-inflammatory effects by activating the Nrf2 signaling pathway and inhibiting NF-κB activation. Moreover, some endogenous aldehydes, including 4-HNE, may serve as signaling molecules involved in cellular senescence and adaptive stress responses [12]. Although previous studies have demonstrated that high concentrations of specific aldehydes, such as glutaraldehyde, can promote vascular calcification in experimental studies [13], the health effects of simultaneous exposure to multiple aldehydes remain largely unexplored, especially at low, environmentally relevant concentrations. Furthermore, the role of endogenously produced aldehydes in AAC has not been comprehensively investigated. Therefore, a thorough understanding of how diverse aldehyde exposures collectively influence AAC is essential for elucidating their contributions to vascular pathology and identifying potential therapeutic targets for preventing calcification-related cardiovascular diseases.

In this study, we performed a population-based analysis using data from the 2013–2014 cycle of the National Health and Nutrition Examination Survey (NHANES) [14]. Specifically, we evaluated the association between AAC and exposure to six commonly detected aldehydes, including benzaldehyde, butyraldehyde, heptanaldehyde, hexanaldehyde, isopentanaldehyde, and propanaldehyde. By examining these aldehydes both individually and as a chemical mixture, our study aims to clarify their collective impact on AAC and provide novel insights into potential strategies for reducing cardiovascular risk associated with vascular calcification.

## Methods

### Study population

This study utilized cross-sectional data from the 2013–2014 cycle of the NHANES, which included a total of 10,175 participants. This cycle was selected because it is the only wave that concurrently measured both AAC and serum aldehyde concentrations. Participants were excluded if they lacked AAC assessment or had missing serum aldehyde data. Key baseline covariates were collected, including demographics (age and sex), smoking status, anthropometric measures (body mass index [BMI], waist-to-height ratio [WHtR]), biochemical indicators (lipid profile, serum calcium, phosphate, creatinine, and klotho), and metabolic comorbidities (hypertension [HBP], diabetes, and hyperuricemia [HUA]). Smoking status was categorized into three groups based on responses to the NHANES questionnaire items SMQ020 and SMQ040: never smokers (SMQ020 = 2), former smokers (SMQ020 = 1 and SMQ040 = 1), and current smokers (SMQ020 = 1 and SMQ040 = 2).

### Measurement of serum aldehydes

The 2013–2014 NHANES cycle measured a total of 12 serum aldehydes. Serum aldehyde concentrations were quantified at the National Center for Environmental Health, Centers for Disease Control and Prevention, using an automated method based on solid phase microextraction (SPME) gas chromatography and high-resolution mass spectrometry with isotope dilution. To accurately measure these highly reactive compounds, the protocol targeted aldehyde-Schiff base protein adducts. Serum samples were automatically incubated with hydrochloric acid (pH ~ 3) to hydrolyze these adducts and release the free aldehydes, which were then captured by a SPME fiber (https://wwwn.cdc.gov/Nchs/Data/Nhanes/Public/2013/DataFiles/ALD_H.htm). This approach minimizes pre-analytical degradation and ensures reproducible measurement of aldehyde levels derived from stable protein adducts. Values below the lower limit of detection (LLOD) were imputed as LLOD divided by the square root of 2 (LLOD/$\surd 2$), in accordance with NHANES standard protocols. Among the 12 measured aldehydes, six with high detection rates were included in the analysis: benzaldehyde, butyraldehyde, heptanaldehyde, hexanaldehyde, isopentanaldehyde, and propanaldehyde (detection: 918 (76.0%), 932 (77.2%), 846 (70.0%), 808 (66.9%), 791 (65.5%), and 959 (79.4%), respectively). The other six aldehydes were excluded due to low detection (< 40%), to minimize imputation burden and reduce potential bias.

### Assessment of AAC

AAC was assessed using lateral lumbar spine dual-energy X-ray absorptiometry scans, performed with the Hologic Discovery A densitometer (Hologic, Inc., Marlborough, MA, USA). The presence and severity of calcification were scored using the validated Kauppila semiquantitative scoring method. AAC was defined as a total Kauppila score ≥ 1, indicating detectable calcification at any lumbar vertebral level (L1–L4). A score of 0 indicated the absence of visible calcification and was classified as non-AAC.

### Statistical analysis

All statistical analyses accounted for the NHANES complex, multistage probability sampling design and incorporated appropriate sample weights to ensure nationally representative estimates. Analyses were conducted using R software version 4.3.2 [15]. To address this missing data while maintaining the complete sample of 1,208 participants, and minimize potential selection bias, we employed multiple imputation by chained equations. We created 5 imputed datasets, incorporating all exposure variables, and key covariates into the imputation model. Consequently, all reported results in the main analysis are based on the multiply imputed data from the full study population. Right-skewed variables, including serum aldehyde concentrations, were log₂-transformed to approximate normality.

Associations between individual aldehyde levels (per log₂-unit increase) and AAC were assessed using weighted bi-variable and multivariable logistic regression. Two models were constructed: Model 1 adjusted for age and sex; Model 2 additionally adjusted for smoking, serum creatinine, klotho, hypertension, and dysglycemia. To account for multiple testing across the six aldehyde exposures, we applied the False Discovery Rate (FDR) correction using the Benjamini–Hochberg procedure. Aldehydes were also analyzed categorically as tertiles (T1–T3), with the lowest tertile (T1) serving as the reference group.

To assess the combined effects of multiple aldehydes, we employed quantile-based g-computation (qgcomp), adjusting for age, sex, smoking, creatinine, klotho, hypertension, and diabetes. Subgroup analyses were further conducted by stratifying participants according to age, sex, renal function (based on creatinine levels), hypertension status, and diabetes status to evaluate the robustness and consistency of the findings. A two-sided *p* < 0.05 was considered statistically significant.

## Results

### Characteristics of the study population

After excluding participants without AAC data (n = 7,035) or serum aldehyde measurements (n = 1,932), a total of 1,208 adults were included in the final analysis. Among them, 859 individuals (73%) did not exhibit AAC. The baseline characteristics of participants with and without AAC are summarized in Table 1. The median age in the AAC group was significantly higher than that in the non-AAC group (63.0 vs. 55.0 years). There were no significant differences between the two groups in terms of sex distribution, smoking status, BMI or WHtR. However, individuals with AAC had higher serum creatinine levels (81.33 µmol/L vs. 77.79 µmol/L) and lower circulating klotho concentrations (743.60 pg/mL vs. 814.10 pg/mL). Additionally, the prevalence of hypertension and diabetes was notably higher in the AAC group compared to those without AAC.

**Table 1:** Clinical characteristics of participants

Characteristic	Total	Non-AAC	AAC	*p* value^b^
	(N = 1,208)	(N = 859)^a^	(N = 349)^a^	
Age (years)	57.00 (48.00, 66.00)	55.00 (46.00, 64.00)	63.00 (54.00, 73.00)	** < 0.001**
Sex^a^				0.357
Male	575 (48.72%)	393 (48.12%)	182 (50.37%)	
Female	633 (51.28%)	466 (51.88%)	167 (449.63%)	
Smoking				0.064
Never	642 (53.13%)	479 (56.40%)	163 (44.07%)	
Former	364 (28.49%)	240 (26.03%)	124 (35.31%)	
Current	202 (18.38%)	140 (17.57%)	62 (20.63%)	
BMI (kg/m^2^)	28.10 (24.90, 32.80)	28.30 (24.90, 33.30)	27.90 (25.00, 31.50)	0.258
WHtR	59.63 (54.05, 66.36)	59.53 (53.58, 66.69)	59.91 (55.02, 65.23)	0.428
Cholesterol (mmol/L)	5.02 (4.29, 5.79)	5.07 (4.29, 5.79)	4.89 (4.24, 5.74)	0.233
Triglycerides (mmol/L)	1.43 (0.94, 2.18)	1.42 (0.90, 2.13)	1.50 (1.10, 2.19)	0.065
Creatinine (µmol/L)	78.68 (66.30, 91.94)	77.79 (65.42, 90.17)	81.33 (68.07, 97.24)	** < 0.001**
Klotho (pg/ml)	792.70 (665.90, 950.40)	814.10 (679.20, 983.30)	743.60 (627.60, 879.00)	**0.010**
Calcium (mmol/L)	2.35 (2.30, 2.43)	2.35 (2.30, 2.40)	2.35 (2.30, 2.43)	0.857
Phosphorus (mmol/L)	1.23 (1.10, 1.36)	1.23 (1.07, 1.36)	1.23 (1.10, 1.36)	0.203
HBP^a^	592 (44.65%)	384 (40.86%)	208 (55.12%)	** < 0.001**
Diabetes^a^				** < 0.001**
Yes	210 (14.07%)	127 (12.11%)	83 (19.49%)	
No	947 (81.66%)	698 (84.06%)	249 (75.00%)	
Borderline	51 (4.28%)	34 (3.83%)	17 (5.51%)	
CKD^a^	52 (3.34%)	30 (2.77%)	22 (4.90%)	0.193
HUA^a^	155 (12.88%)	104 (12.79%)	51 (13.11%)	0.887

*AAC* Abdominal aortic calcification, *BMI* Body mass index, *CKD* Chronic kidney disease, *HBP* Hypertension, *HUA* Hyperuricemia, *WHtR* Waist-to-height ratio

^a^n (unweighted) (%)

^b^*p*-values derived from weighted Pearson’s chi-square test (categorical variables) or quantile regression (continuous variables). Bold values indicate that are statistically significant at *p* < 0.05

### Association between individual aldehydes and AAC

We initially explored the relationship between individual aldehyde levels and AAC. Serum butyraldehyde levels were significantly lower in the AAC group than in the non-AAC group (median: −0.77 vs. −0.67; *p* = 0.017 (Table 2). Other aldehydes, including benzaldehyde, heptanaldehyde, hexanaldehyde, isopentanaldehyde, and propanaldehyde, did not differ significantly between groups (all *p* > 0.1).

**Table 2:** Serum aldehyde levels by AAC status

Characteristic^a^	Total	Non-AAC	AAC	*p* value
	(N = 1,208)	(N = 859)^a^	(N = 349)^a^	
Benzaldehyde	0.31 (−0.09, 0.71)	0.33 (−0.07, 0.72)	0.26 (−0.23, 0.68)	0.187
Butyraldehyde	−0.69 (−1.04, −0.40)	−0.67 (−0.99, −0.39)	−0.77 (−1.16, −0.47)	0.017
Heptanaldehyde	−0.71 (−0.88, −0.54)	−0.71 (−0.88, −0.54)	−0.76 (−0.92, −0.53)	0.373
Hexanaldehyde	0.74 (0.53, 0.93)	0.74 (0.53, 0.93)	0.74 (0.51, 0.97)	0.973
Isopentanaldehyde	−0.84 (−1.13, −0.45)	−0.85 (−1.14, −0.46)	−0.82 (−1.12, −0.44)	0.807
Propanaldehyde	0.64 (0.37, 0.87)	0.64 (0.36, 0.88)	0.63 (0.37, 0.86)	0.703

^a^Aldehyde concentrations: Log-transformed values (natural logarithm)

To further assess the association, we performed weighted bi-variable and multivariable logistic regression analyses. In the fully adjusted model (Model 2), which accounted for age, sex, smoking, creatinine, klotho, hypertension, and dysglycemia, each log₂-unit increase in serum butyraldehyde was associated with a 36.7% reduction in the odds of AAC (adjusted OR = 0.633; 95% CI 0.436–0.930; *p* = 0.016) (Table 3). In contrast, none of the other aldehydes showed significant associations with AAC in fully adjusted models (all *p* > 0.25; Table 3). After FDR correction for multiple comparisons, butyraldehyde showed the strongest association with AAC (FDR-adjusted *p* = 0.096).

**Table 3:** Bi-variable and multivariable logistic regression analysis of aldehydes and AAC

	Crude model		Model 1		Model 2		FDR-adjust
	OR (95% CI)	*p* value	OR (95% CI)	*p* value	OR (95% CI)	*p* value	*p* value
Benzaldehyde	0.810 (0.585–1.121)	0.186	0.797 (0.554–1.146)	0.199	0.823 (0.530–1.210)	0.251	0.704
Butyraldehyde	0.662 (0.461–0.950)	0.028	0.683 (0.479–0.975)	0.038	0.633 (0.436–0.930)	0.016	0.096
Heptanaldehyde	0.740 (0.372–1.469)	0.362	0.838 (0.392–1.789)	0.620	0.893 (0.384–2.073)	0.744	0.744
Hexanaldehyde	1.166 (0.647–2.099)	0.585	1.306 (0.740–2.307)	0.326	1.376 (0.618–3.061)	0.352	0.704
Isopentanaldehyde	1.025 (0.678–1.551)	0.899	1.142 (0.710–1.836)	0.555	0.918 (0.565–1.489)	0.667	0.744
Propanaldehyde	1.102 (0.703–1.729)	0.650	1.242 (0.791–1.951)	0.315	1.139 (0.722–1.795)	0.495	0.742

*AAC* Abdominal aortic calcification, *CI* Confidence interval, *OR* Odds ratio

Crude: Unadjusted; Model 1: Adjusted for sex and age; Model 2: Adjusted for sex, age, smoking, creatinine, klotho, hypertension, and dysglycemia

Aldehyde concentrations: Log-transformed values (natural logarithm)

The FDR-adjusted* p* values were calculated using the Benjamini–Hochberg procedure on the original *p* values from Model 2.

A dose–response relationship was observed in the tertile analysis of log-transformed butyraldehyde levels: compared to the lowest tertile (T1), participants in the highest tertile (T3) had a 46% lower risk of AAC (adjusted OR = 0.542; 95% CI 0.363–0.811; p = 0.013) (Table 4). These findings suggest a unique inverse association between butyraldehyde and AAC, whereas other aldehydes showed no significant association.

**Table 4:** Logistic regression analysis for the association between butyraldehyde and AAC

	Crude model		Model 1		Model 2	
	OR (95% CI)	*p* value	OR (95% CI)	*p* value	OR (95% CI)	*p* value
Total	0.662 (0.461–0.950)	0.028	0.683 (0.479–0.975)	0.038	0.633 (0.436–0.930)	**0.016**
T1	1.000	–	1.000	–	1.000	–
T2	0.696 (0.463–1.046)	0.077	0.729 (0.493–1.078)	0.103	0.741 (0.461–1.192)	0.155
T3	0.574 (0.407–0.810)	0.004	0.590 (0.427–0.815)	0.004	0.542 (0.363–0.811)	**0.013**

T1/T2/T3: Tertiles of butyraldehyde concentration (T1: lowest, T3: highest)

Crude: Unadjusted; Model 1: Adjusted for sex and age; Model 2: Adjusted for sex, age, smoking, creatinine, klotho, hypertension status, and diabetes status.  Bold values indicate that are statistically significant at *p* < 0.05

### Joint effects of mixed aldehyde exposure on AAC: Qgcomp model analysis

**Fig. 1:**
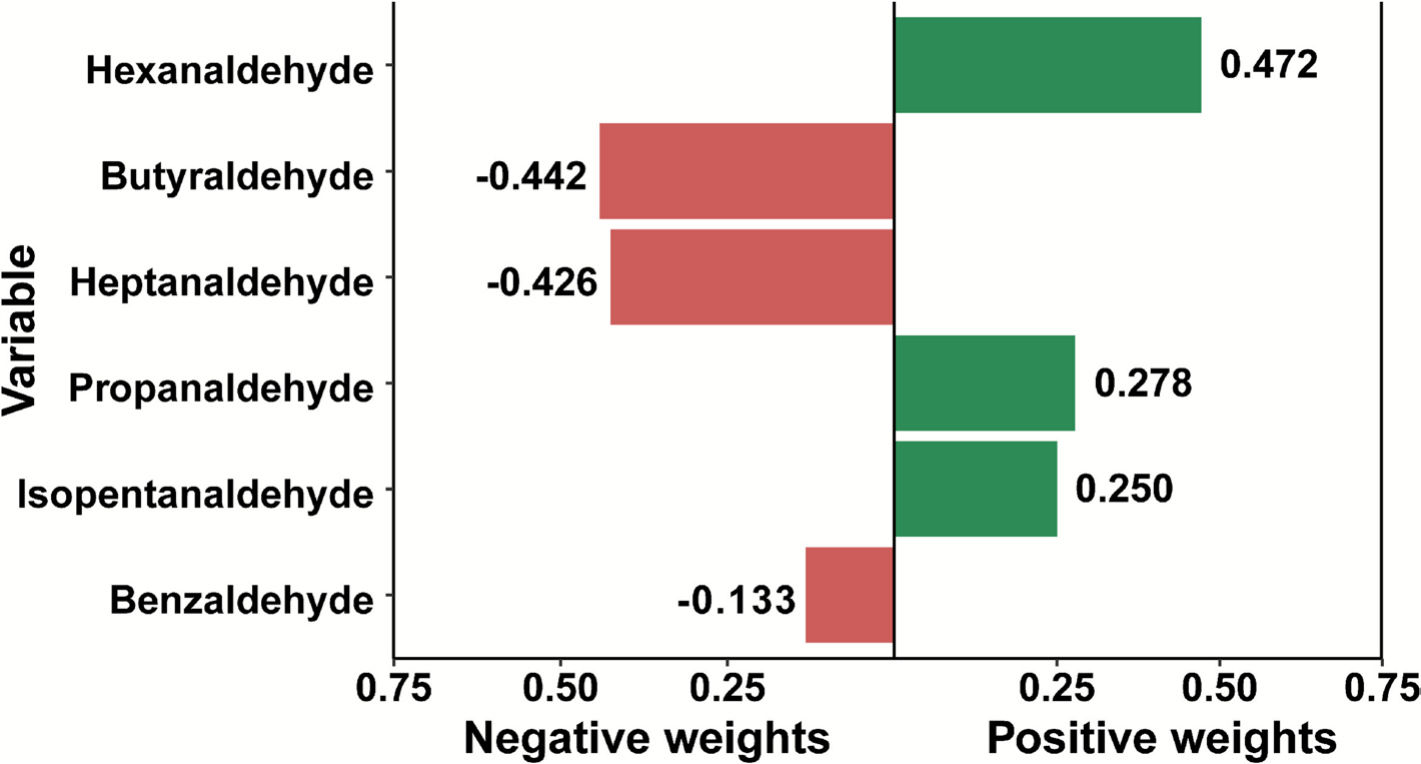
Weighted contributions of individual aldehydes to AAC risk based on qgcomp analysis. Bars represent the relative negative and positive weights of six aldehydes in their joint association with AAC. The model adjusts for age, sex, smoking, creatinine, klotho, hypertension status, and diabetes status

To assess the combined impact of multiple aldehyde exposures, we employed qgcomp. The analysis demonstrated a significant inverse association between total aldehyde mixture and AAC risk, with butyraldehyde contributing the largest negative weight (β = −0.442), indicating it was the dominant contributor to the overall inverse association (Fig. 1).

### Subgroup analysis: inverse association of klotho with AAC

Subgroup analyses were performed by stratifying participants based on key characteristics. The sample sizes are as follows: Klotho (High: n = 603, Low: n = 605), age (< 65 years: n = 801, ≥ 65 years: n = 407), renal function by creatinine level (Cr < 133 µmol/L: n = 1165, Cr ≥ 133 µmol/L: n = 43), and diabetes status (No diabetes: n = 947, Diabetes: n = 210, Borderline: n = 51). All statistical analyses, including the interaction tests and odds ratio estimates, incorporated the complex NHANES survey weights to provide nationally representative estimates.

**Fig. 2:**
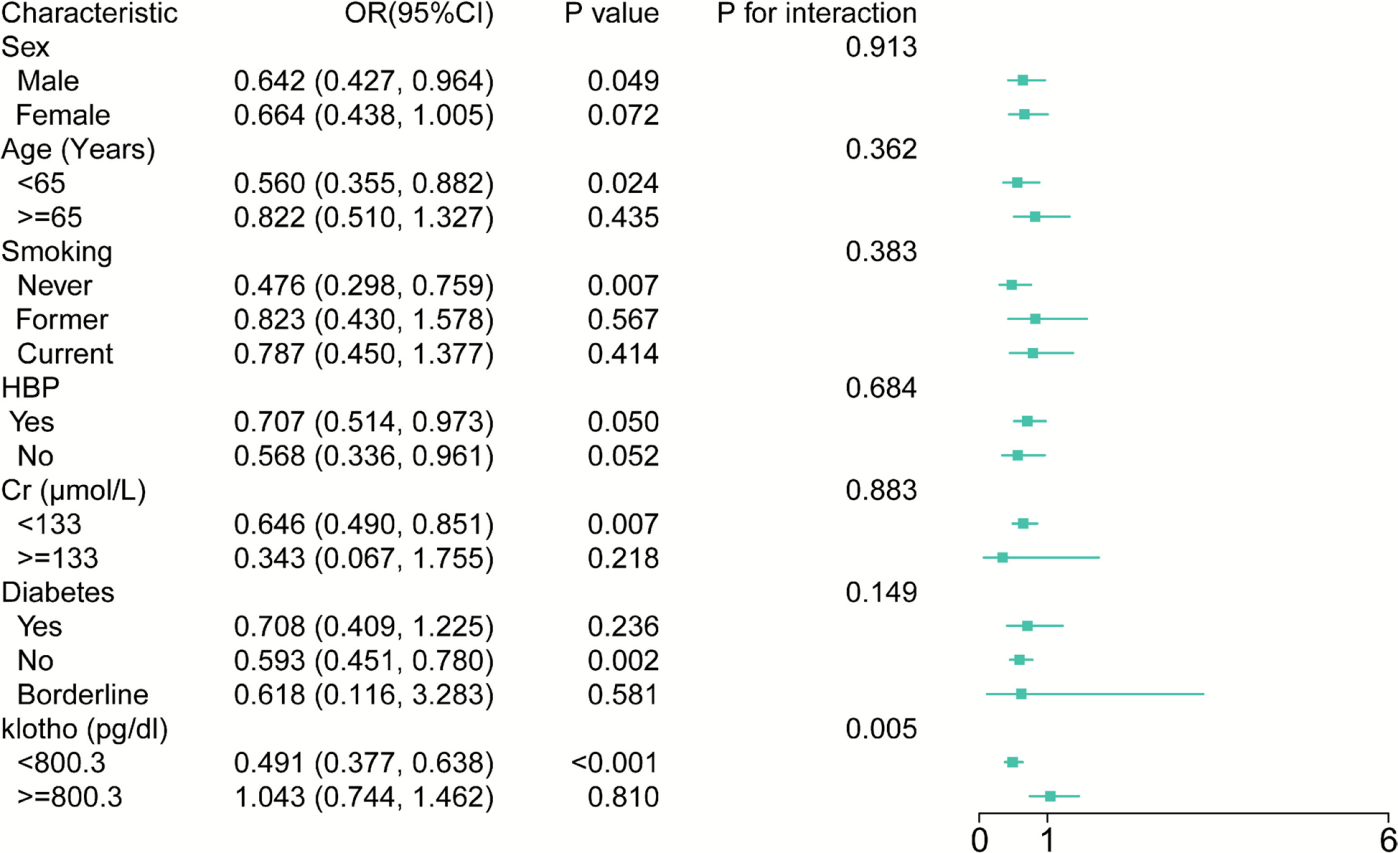
Stratified analysis of the association between butyraldehyde and AAC. *AAC* abdominal aortic calcification, *CI* confidence interval, *Cr* creatinine, *HBP* hypertension, *OR* odds ratio

Subgroup analyses indicated that higher circulating klotho levels (≥ 800.3 pg/mL, median split) were significantly associated with reduced AAC risk. Individuals in the high-klotho group had 51% lower odds of AAC compared to those with lower klotho levels (OR = 0.491; 95% CI 0.377–0.6638; *p* < 0.0001). A statistically significant interaction was observed between klotho concentration and AAC risk (*p*-interaction = 0.005). Furthermore, stratified analyses showed stronger inverse associations between butyraldehyde and AAC among younger adults (< 65 years) and those without diabetes, suggesting that the relationship may be influenced by age and metabolic health status (Fig. 2).

## Discussion

Aldehyde exposure has been implicated in the pathogenesis of various diseases [16, 17], raising increasing concern for public health. Our study provides novel insights into the complex roles of serum aldehydes in AAC. Utilizing data from a nationally representative population, we identified butyraldehyde as the first aldehyde to demonstrate a significant inverse dose–response relationship with AAC. This inverse association was consistently observed across demographic and metabolic subgroups. This finding was further confirmed through mixture modeling, which identified butyraldehyde as the primary factor involved in the development of AAC. While the association between butyraldehyde and AAC was attenuated after rigorous FDR correction for multiple comparisons, it remained the most consistent signal among all aldehydes examine. Notably, this inverse association differed markedly from the positive correlation observed for structural analogs like isopentanaldehyde with calcification, suggesting that specific physicochemical properties of individual aldehydes may influence their relationships with vascular outcomes. Our findings provide evidence against categorizing all aldehydes as uniformly harmful to vascular health. This work supports future research on butyraldehyde and related compounds to explore their potential role in vascular calcification.

Aldehydes display contrasting effects in physiological environments, with both pathological consequences and therapeutic benefits. For example, formaldehyde remains widely employed as a disinfectant and tissue fixative in laboratory and clinical practice [18]. Similarly, cinnamaldehyde exhibits valuable biological activities including antioxidant, antimicrobial, and anti-obesity effects [19]. Conversely, endogenous aldehydes generated through lipid peroxidation, such as 4-HNE, have emerged as key biomarkers and mediators of oxidative stress-related disorders, including diabetes and cancer [9]. Within biomedical engineering, glutaraldehyde has long been utilized in the fabrication of bioprosthetic heart valves due to its capacity to enhance mechanical strength and inhibit biodegradation [20]. However, its use is limited by a tendency to promote calcification, which represents a major limitation for the long-term function of these medical devices.

Our findings further highlight the vascular implications of aldehyde exposure. Although isopentanaldehyde, which shares structural similarity with glutaraldehyde, showed a nonsignificant association with AAC risk (OR = 0.918, *p* = 0.667), butyraldehyde showed a statistically significant inverse association (OR = 0.633, *p* = 0.016). This pattern was observed consistently in both individual regression and mixture models. The observed inverse association between butyraldehyde and AAC presents a notable contrast to the established pro-oxidant and cytotoxic effects of many aldehydes. This discrepancy may be explained by differences in biochemical reactivity: glutaraldehyde and related compounds are known to induce cytotoxicity and disrupt extracellular matrix via excessive protein cross-linking [21, 22], thereby promoting calcium deposition. In contrast, butyraldehyde derivatives such as 2,3-butanediol have shown more favorable biocompatibility, including reduced platelet adhesion and lower calcification potential [23]. These beneficial effects may result from their ability to stabilize collagen without activating pro-inflammatory or pro-oxidant cascades. Our discovery for butyraldehyde provides additional evidence for the understanding that specific aldehydes can serve as beneficial signaling molecules or metabolic intermediates, not just as damaging agents. This supports the potential development of short-chain aldehydes with moderate reactivity for biomedical applications. Such compounds could lead to improved tissue stabilizers that maintain structural integrity while reducing calcification.

In addition to their effects on extracellular matrix stability, aldehydes may also modulate vascular calcification through oxidation–reduction signaling pathways, such as those involving the Klotho-FGF23 axis [24]. Klotho, a circulating anti-aging protein, plays a central role in phosphate homeostasis and the inhibition of osteogenic transformation in vascular smooth muscle cells. Experimental studies have shown that Klotho suppresses vascular smooth muscle cells calcification by enhancing FGF23 signaling, reducing oxidative stress, and dampening inflammatory responses [24]. Our findings of a significant interaction between serum Klotho and butyraldehyde (*p*-interaction = 0.005) suggest a potential compensatory mechanism. In individuals with Klotho deficiency (< 800.3 pg/mL), higher butyraldehyde levels were strongly associated with reduced AAC risk (OR = 0.491, 95% CI 0.377–0.638). This interaction supports the known behavior of aldehydes like 4-HNE, which, at low levels, upregulate cytoprotective pathways but can become toxic at higher concentrations [25]. We hypothesize that Klotho deficiency may enable butyraldehyde to exert a compensatory role by restoring antioxidant signaling. However, in Klotho-sufficient individuals, butyraldehyde might have limited benefit association and could even manifest its toxic potential. Further research is needed to clarify the molecular interactions between butyraldehyde and the Klotho-FGF23 pathway.

Taken together, these findings highlight the independent role of butyraldehyde against vascular calcification, even in the presence of other aldehydes, suggesting its importance in the overall aldehyde burden. The question remains whether this relationship is mediated by butyraldehyde itself or its metabolite, butyrate, a short-chain fatty acid with known anti-inflammatory properties [26]. While exogenous butyrate supplementation can raise circulating levels, butyrate derived from gut microbiota or hepatic conversion of butyraldehyde undergoes significant first-pass metabolism, limiting its systemic bioavailability [27, 28]. In contrast, butyraldehyde, being a small, volatile compound, can readily diffuse across biological membranes. Considering these metabolic characteristics, along with our mixture analysis identifying butyraldehyde as the most significant inverse predictor, the observed association with aortic calcification is more likely attributed to butyraldehyde itself rather than to its metabolic conversion to butyric acid.

The observed association between butyraldehyde and reduced aortic calcification, derived from a large, nationally representative sample with standardized biomarker measurements, offers new epidemiological evidence. However, several limitations must be acknowledged. Although the cross-sectional design of our data does not allow us to establish causal conclusions, it successfully revealed a previously unreported relationship, creating a clearly hypothesis for future longitudinal studies. While potential confounding factors, such as dietary habits and specific inflammatory markers (e.g., C-reactive protein), were not available in the NHANES 2013–2014 cycle, our analysis carefully adjusted for numerous established cardiometabolic risk factors including smoking, diabetes, and hypertension, and the association remained significant. Finally, the lack of butyric acid measurements prevented formal mediation analysis; however, this limitation necessitated a reasoned interpretation based on metabolic characteristics. The specific association of butyraldehyde, unlike other aldehydes, supports our interpretation of a direct effect based on the extensive hepatic clearance of butyrate, and confirms the strength of our findings. To address these limitations and build on our findings, future studies should: (1) validate the epidemiological evidence in multi-ethnic cohorts to assess generalizability and temporality; (2) explore the molecular mechanisms by integrating aldehyde profiling with multi-omics approaches in relevant models; (3) conduct in vitro and in vivo experiments to investigate the effects of butyraldehyde on vascular smooth muscle cells; and (4) clarify the origins of exposure to differentiate endogenous versus exogenous contributions to serum butyraldehyde levels.

## Conclusions

In summary, our population-based analysis identifies a significant inverse association between serum butyraldehyde and AAC that persists after accounting for conventional risk factors. This evidence identifies butyraldehyde as a promising candidate for further research, providing an epidemiological basis for future studies into its underlying mechanisms and potential therapeutic applications.

## Data Availability

No datasets were generated or analysed during the current study.

## Abbreviations

4-HNE: 4-Hydroxynonenal
AAC: Abdominal aortic calcification
BMI: Body mass index
CI: Confidence interval
CKD: Chronic kidney disease
Cr: Creatinine
FDR: False discovery rate
HBP: Hypertension
HUA: Hyperuricemia
LLOD: Lower limit of detection
OR: Odds ratio
NHANES: National Health and Nutrition Examination Survey
qgcomp: Quantile g-computation
SPME: Solid phase microextraction
WHtR: Waist-to-height ratio

## References

[1] Leow K, Szulc P, Schousboe JT, Kiel DP, Teixeira-Pinto A, Shaikh H, et al. Prognostic value of abdominal aortic calcification: a systematic review and meta-analysis of observational studies. J Am Heart Assoc. 2021;10(2):e017205. 33439672 10.1161/JAHA.120.017205 PMC7955302

[2] Schousboe JT, Lewis JR, Kiel DP. Abdominal aortic calcification on dual-energy X-ray absorptiometry: methods of assessment and clinical significance. Bone. 2017;104:91–100. 28119178 10.1016/j.bone.2017.01.025

[3] Radavelli-Bagatini S, Bondonno CP, Dalla Via J, Sim M, Gebre AK, Blekkenhorst LC, et al. Impact of provision of abdominal aortic calcification results on fruit and vegetable intake: 12-week randomized phase 2 controlled trial. Nat Commun. 2024;15(1):8126. 39402045 10.1038/s41467-024-52172-1 PMC11473756

[4] Ahmed Laskar A, Younus H. Aldehyde toxicity and metabolism: the role of aldehyde dehydrogenases in detoxification, drug resistance and carcinogenesis. Drug Metab Rev. 2019;51(1):42–64. 30514131 10.1080/03602532.2018.1555587

[5] Zhu Z, Lu J, Wang S, Peng W, Yang Y, Chen C, et al. Acrolein, an endogenous aldehyde induces synaptic dysfunction in vitro and in vivo: involvement of RhoA/ROCK2 pathway. Aging Cell. 2022;21(4):e13587. 35315217 10.1111/acel.13587 PMC9009232

[6] Di Domenico F, Tramutola A, Butterfield DA. Role of 4-hydroxy-2-nonenal (HNE) in the pathogenesis of alzheimer disease and other selected age-related neurodegenerative disorders. Free Radic Biol Med. 2017;111:253–61. 27789292 10.1016/j.freeradbiomed.2016.10.490

[7] Zhu R, Liu H, Liu C, Wang L, Ma R, Chen B, et al. Cinnamaldehyde in diabetes: a review of pharmacology, pharmacokinetics and safety. Pharmacol Res. 2017;122:78–89. 28559210 10.1016/j.phrs.2017.05.019

[8] Maessen DEM, Stehouwer CDA, Schalkwijk CG. The role of methylglyoxal and the glyoxalase system in diabetes and other age-related diseases. Clin Sci. 2015;128(12):839–61. 10.1042/CS20140683 25818485

[9] Jaganjac M, Zarkovic N. Lipid peroxidation linking diabetes and cancer: the importance of 4-hydroxynonenal. Antioxid Redox Signal. 2022;37(16–18):1222–33. 36242098 10.1089/ars.2022.0146

[10] Han R, Li X, Gao X, Lv G. Cinnamaldehyde: pharmacokinetics, anticancer properties and therapeutic potential (Review). Mol Med Rep. 2024;30(3):163. 38994757 10.3892/mmr.2024.13287 PMC11267250

[11] Cui X, Zhang L, Lin L, Hu Y, Zhang M, Sun B, et al. Notoginsenoside R1-Protocatechuic aldehyde reduces vascular inflammation and calcification through increasing the release of nitric oxide to inhibit TGFβR1-YAP/TAZ pathway in vascular smooth muscle cells. Int Immunopharmacol. 2024;143(Pt 3):113574. 39520961 10.1016/j.intimp.2024.113574

[12] Monroe TB, Hertzel AV, Dickey DM, Hagen T, Santibanez SV, Berdaweel IA, et al. Lipid peroxidation products induce carbonyl stress, mitochondrial dysfunction, and cellular senescence in human and murine cells. Aging Cell. 2025;24(1):e14367. 39394673 10.1111/acel.14367 PMC11709094

[13] Zilla P, Weissenstein C, Bracher M, Zhang Y, Koen W, Human P, et al. High glutaraldehyde concentrations reduce rather than increase the calcification of aortic wall tissue. J Heart Valve Dis. 1997;6(5):502–9. 9330172

[14] CDC. National Health and Nutrition Examination Survey. 2025. https://www.cdc.gov/nchs/nhanes/index.html. Accessed 02 Jun 2025.

[15] R: The R Project for Statistical Computing. https://www.r-project.org/. Accessed 02 Jun 2025.

[16] Griffiths SD, Entwistle JA, Kelly FJ, Deary ME. Characterising the ground level concentrations of harmful organic and inorganic substances released during major industrial fires, and implications for human health. Environ Int. 2022;162:107152. 35231840 10.1016/j.envint.2022.107152

[17] Vijayraghavan S, Saini N. Aldehyde-Associated Mutagenesis─Current State of Knowledge. Chem Res Toxicol. 2023;36(7):983–1001. 37363863 10.1021/acs.chemrestox.3c00045 PMC10354807

[18] Soltanpour Z, Mohammadian Y, Fakhri Y. The exposure to formaldehyde in industries and health care centers: a systematic review and probabilistic health risk assessment. Environ Res. 2022;204(Pt B):112094. 34563530 10.1016/j.envres.2021.112094

[19] Muhoza B, Qi B, Harindintwali JD, Koko MYF, Zhang S, Li Y. Encapsulation of cinnamaldehyde: an insight on delivery systems and food applications. Crit Rev Food Sci Nutr. 2023;63(15):2521–43. 34515594 10.1080/10408398.2021.1977236

[20] Chang J, Yu L, Lei J, Liu X, Li C, Zheng Y, et al. A multifunctional bio-patch crosslinked with glutaraldehyde for enhanced mechanical performance, anti-coagulation properties, and anti-calcification properties. J Mater Chem B. 2023;11(43):10455–63. 37888984 10.1039/d3tb01724a

[21] Golomb G, Schoen FJ, Smith MS, Linden J, Dixon M, Levy RJ. The role of glutaraldehyde-induced cross-links in calcification of bovine pericardium used in cardiac valve bioprostheses. Am J Pathol. 1987;127(1):122–30. 3105321 PMC1899585

[22] Sinha P, Zurakowski D, Kumar TKS, He D, Rossi C, Jonas RA. Effects of glutaraldehyde concentration, pretreatment time, and type of tissue (porcine versus bovine) on postimplantation calcification. J Thorac Cardiovasc Surg. 2012;143(1):224–7. 22047684 10.1016/j.jtcvs.2011.09.043

[23] Ren K, Duan W, Liang Z, Yu B, Li B, Jin Z, et al. Glutaraldehyde and 2,3-butanediol treatment of bovine pericardium for aortic valve bioprosthesis in sheep: a preliminary study. Ann Transl Med. 2020;8(24):1668. 33490180 10.21037/atm-20-7803 PMC7812161

[24] Urakawa I, Yamazaki Y, Shimada T, Iijima K, Hasegawa H, Okawa K, et al. Klotho converts canonical FGF receptor into a specific receptor for FGF23. Nature. 2006;444(7120):770–4. 17086194 10.1038/nature05315

[25] Jaganjac M, Milkovic L, Zarkovic N, Zarkovic K. Oxidative stress and regeneration. Free Radic Biol Med. 2022;181:154–65. 35149216 10.1016/j.freeradbiomed.2022.02.004

[26] Mann ER, Lam YK, Uhlig HH. Short-chain fatty acids: linking diet, the microbiome and immunity. Nat Rev Immunol. 2024;24(8):577–95. 38565643 10.1038/s41577-024-01014-8

[27] Prins GH, Rios-Morales M, Gerding A, Reijngoud DJ, Olinga P, Bakker BM. The effects of butyrate on induced metabolic-associated fatty liver disease in precision-cut liver slices. Nutrients. 2021;13(12):4203. 34959755 10.3390/nu13124203 PMC8703944

[28] Stoeva MK, Garcia-So J, Justice N, Myers J, Tyagi S, Nemchek M, et al. Butyrate-producing human gut symbiont, *Clostridium butyricum*, and its role in health and disease. Gut Microbes. 2021;13(1):1–28. 33874858 10.1080/19490976.2021.1907272 PMC8078720

